# Reduced risk of hospitalisation among reported COVID-19 cases infected with the SARS-CoV-2 Omicron BA.1 variant compared with the Delta variant, Norway, December 2021 to January 2022

**DOI:** 10.2807/1560-7917.ES.2022.27.4.2200077

**Published:** 2022-01-27

**Authors:** Lamprini Veneti, Håkon Bøås, Anja Bråthen Kristoffersen, Jeanette Stålcrantz, Karoline Bragstad, Olav Hungnes, Margrethe Larsdatter Storm, Nina Aasand, Gunnar Rø, Jostein Starrfelt, Elina Seppälä, Reidar Kvåle, Line Vold, Karin Nygård, Eirik Alnes Buanes, Robert Whittaker

**Affiliations:** 1 Department of Infection Control and Preparedness, Norwegian Institute of Public Health, Oslo, Norway; 2 Department of Infection Control and Vaccines, Norwegian Institute of Public Health, Oslo, Norway; 3 Department of Method Development and Analytics, Norwegian Institute of Public Health, Oslo, Norway; 4 European Program for Intervention Epidemiology Training, European Centre for Disease Prevention and Control, Stockholm, Sweden; 5 Department of Virology, Norwegian Institute of Public Health, Oslo, Norway; 6 Department of Infectious Disease Registries, Norwegian Institute of Public Health, Oslo, Norway; 7 Department of Anaesthesia and Intensive Care, Haukeland University Hospital, Bergen, Norway; 8 Department of Clinical Medicine, University of Bergen, Bergen, Norway; 9 Norwegian Intensive Care and Pandemic Registry, Haukeland University Hospital, Bergen, Norway

**Keywords:** Norway, COVID-19, B.1.1.529, Omicron, Delta, hospitalisation

## Abstract

We included 39,524 COVID-19 Omicron and 51,481 Delta cases reported in Norway from December 2021 to January 2022. We estimated a 73% reduced risk of hospitalisation (adjusted hazard ratio: 0.27; 95% confidence interval: 0.20–0.36) for Omicron compared with Delta. Compared with unvaccinated groups, Omicron cases who had completed primary two-dose vaccination 7–179 days before diagnosis had a lower reduced risk than Delta (66% vs 93%). People vaccinated with three doses had a similar risk reduction (86% vs 88%).

In Norway, the first coronavirus disease (COVID-19) cases infected with the severe acute respiratory syndrome coronavirus 2 (SARS-CoV-2) variant Omicron (Phylogenetic Assignment of Named Global Outbreak Lineages (Pangolin) designation B.1.1.529) substrain BA.1 were detected in an outbreak following a social gathering on 26 November 2021 where the suspected index case had reported recent travel to South Africa [1]. Since then, testing activity has been high [2,3], and the proportion of cases screened for variant weekly ranged from 49 to 67% up to early January 2022. Omicron became the dominant circulating variant in late December 2021 (Figure 1).

**Figure 1:**
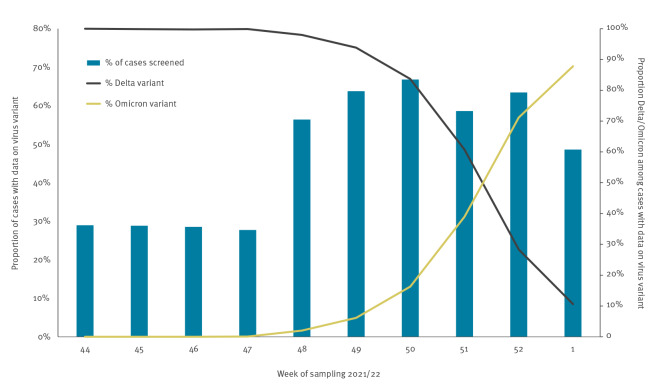
Proportion of reported COVID-19 cases with data on virus variant, and proportion of Delta and Omicron by week of sampling, Norway, 1 November 2021–9 January 2022 (n = 239,391)

COVID-19: coronavirus disease.

Testing activity and screening of cases was enhanced after the detection of the first Omicron (B.1.1.529) cases in Norway (week 48). Variants other than Delta (B.1.617.2) and Omicron were infrequently detected during the study period (week 49–1). During the study period, 155,388 cases were reported and 91,772 (59%) were screened for variants. Of those 91,772, 55% were Delta, 44% Omicron BA.1 and 0.3% Omicron BA.2 (n = 242 in week 1).

We used linked individual-level data from the Norwegian Preparedness Registry (Beredt C19) [4] to estimate the risk of hospitalisation for reported Omicron cases compared with Delta (B.1.617.2) cases. We also compared the length of hospital stay (LoS) and estimated the risk of admission to an intensive care unit (ICU) among hospitalised Omicron and Delta cases, and described deaths in the study cohort.

## Study population

We analysed cases of COVID-19 with a date of positive test between 6 December 2021 and 9 January 2022. We extracted data from Beredt C19 on 20 January 2022, allowing 10 days of follow-up since last positive test. Further details on the data sources and definitions can be found in the Supplement, section 1. Of 158,561 cases reported in the study period, 155,388 (98%) had a national identity number. The national identity number was essential to link data from all registries used in the analysis. Of these 155,388, 91,772 (59%) were screened for variants. Variants were identified based on whole genome sequencing, Sanger partial S-gene sequencing or PCR screening targeting specific single nucleotide polymorphisms, insertions or deletions that reliably differentiate between Omicron and other variants. During the study period, 11% of variants were sequenced. 

We assessed the representativeness of the 91,772 screened cases among the 155,388 reported cases. The proportion of cases screened among hospitalised cases was higher than among non-hospitalised cases (73% vs 59%). We expect this difference to have a limited impact on our estimates, given the small proportion of all cases admitted to hospital, and that screening of hospitalised cases did not depend on variant exposure. Section 2 of the Supplement provides a detailed comparison of screened and not-screened cases.

We only included cases infected with SARS-CoV-2 variants Omicron (excluding all 242 sub-variant BA.2 cases that were reported in week 1) and Delta. Our main outcome was hospitalisation ≤ 2 days before and ≤ 28 days following a positive COVID-19 test, where COVID-19 was the reported main cause of admission. To avoid bias, we excluded 301 Omicron and Delta cases hospitalised with another or unknown main cause of admission.

Overall, 39,524 Omicron (43%) and 51,481 (57%) Delta cases were included. See Table 1 for characteristics of our retrospective study cohort. The median time since last vaccination dose was 201 days (interquartile range (IQR): 190–216; maximum: 316) for cases who had completed primary vaccination with maximum two doses ≥ 180 days before positive test, and 22 days (IQR: 12–36; maximum: 192) for cases vaccinated with three doses.

**Table 1:** Distribution of COVID-19 cases in the retrospective study cohort, by detected variant, for different characteristics and proportion hospitalised, Norway, 6 December 2021–9 January 2022 (n = 91,005)

Characteristic	Study cohort		Variant type (% by characteristic)					Hospitalised cases (% of diagnosed cases)						
			Delta			Omicron		Delta		Omicron		Total		
	n	%	n		%	n	%	n	%	n	%	n		%
Total	91,005	100	51,481		100	39,524	100	552	100	91	100	643		100
Sex														
Female	45,262	50	25,577		50	19,685	50	228	41	53	58	281		44
Male	45,743	50	25,904		50	19,839	50	324	59	38	42	362		56
Age group (years)														
0–29	45,773	50	25,606	50		20,167	51	36	6.5	11	12	47	7.3	
30–44	23,752	26	13,496	26		10,256	26	95	17	14	15	109	17	
45–54	11,656	13	6,537	13		5,119	13	80	14	18	20	98	15	
55–64	6,334	7.0	3,623	7.0		2,711	6.9	116	21	17	19	133	21	
65–74	2,368	2.6	1,549	3.0		819	2.1	96	17	11	12	107	17	
≥ 75	1,122	1.2	670	1.3		452	1.1	129	23	20	22	149	23	
Country of birth														
Norway	66,488	73	38,639	75		27,849	70	323	59	52	57	375	58	
Overseas	23,568	26	12,246	24		11,322	29	203	37	34	37	237	37	
Unknown	949	1.0	596	1.1		353	0.9	26	4.7	5	5.5	31	4.8	
Risk for severe COVID-19^a^														
No underlying comorbidities	81,036	89	45,492	88		35,544	90	297	54	36	40	333	52	
Medium-risk comorbidity	8,667	10	5,159	10		3,508	8.9	145	26	31	34	176	27	
High-risk comorbidity	1,302	0.9	830	1.6		472	1.2	110	20	24	26	134	21	
Vaccination status at date of positive test														
Not vaccinated	30,546	34	22,837	44		7,709	20	335	61	15	16	350	34	
One dose < 21 days before positive test	517	0.6	355	0.7		162	0.4	8	1.5	1	1.1	9	1.4	
Partially completed primary vaccination series ≥ 21 days before positive test^b^	6,507	7.2	3,935	7.6		2,572	6.5	9	1.6	3	3.3	12	1.9	
Completed primary vaccination series with maximum two doses 7–179 days before positive test^b^	39,821	44	17,981	35		21,840	55	47	8.5	26	29	73	11	
Completed primary vaccination series with maximum two doses ≥ 180 days before positive test^b^	8,351	9.2	4,790	9.3		3,561	9.0	98	18	26	29	124	19	
Vaccinated with three doses ≥ 7 days before positive test^b^	4,848	5.3	1,505	2.9		3,343	8.5	55	10	20	22	75	12	
Unvaccinated, but previously diagnosed with COVID-19 6–12 months before positive test	415	0.5	78	0.2		337	0.9	0	0.0	0	0.0	0	0.0	

COVID-19: coronavirus disease.

^a^ Risk for severe disease based on underlying comorbidities that are associated with a moderate or high risk of serious illness regardless of age. Details on the definitions used are provided in the Supplement, section 1.

^b^ See Supplement section 1 for full details on how each vaccination status category is defined and data on vaccine type. Overall, 98% of vaccinated Omicron cases and 96% of Delta cases had received a homologous or mixed regimen of mRNA vaccines. The three-dose category predominantly includes cases who received their third dose as a booster dose, however it will also include cases who received their third dose as part of their primary series, for example those severely immunocompromised [14]. We were not able to clearly distinguish persons who had received a third dose as part of their primary series from those who had received a booster dose.

## Hospitalisation

Overall, 91 (0.2%) Omicron and 552 (1.1%) Delta cases were hospitalised. The median time from positive test to admission was 1 day (IQR: 0–3) for Omicron and 4 days (IQR: 0–7) for Delta.

Using stratified Cox proportional hazard regression, we estimated the risk (adjusted hazard ratio (aHR)) of hospitalisation for infection with the Omicron compared with the Delta variant. The models were stratified by county of residence and sampling date and further adjusted for age group, sex, country of birth, underlying comorbidities and vaccination status. In this main analysis, Omicron was associated with an overall 73% reduced risk of hospitalisation (aHR = 0.27; 95% confidence interval (CI): 0.20–0.36) compared with Delta (Table 2). In section 3 of the Supplement we present detailed information on the statistical analysis and hazard ratio estimates for hospitalisation from univariate and multivariable Cox regression for this analysis.

**Table 2:** Hazard ratio estimates for hospitalisation with the Omicron compared with the Delta variant of SARS-CoV-2, using stratified Cox regression, overall and by subgroup analysis, Norway, 6 December 2021–9 January 2022 (n = 91,005)

	Hospitalisation				
	Delta cases		Omicron cases		Omicron vs Delta, adjusted hazard ratio (95%CI)
	n	% ^a^	n	% ^a^	
Overall/main analysis	552	1.1	91	0.2	0.27 (0.20–0.36)
Subgroup analysis by					
Sex					
Female	228	0.9	53	0.3	0.45 (0.30–0.67)
Male	324	1.3	38	0.2	0.17 (0.11–0.27)
Age group (years)					
0–29	36	0.1	11	0.1	0.24 (0.09–0.60)
30–44	95	0.7	14	0.1	0.23 (0.11–0.47)
45–54	80	1.2	18	0.4	0.40 (0.19–0.85)
55–64	116	3.2	17	0.6	0.22 (0.10–0.45)
65–74	96	6.2	11	1.3	0.20 (0.07–0.51)
≥ 75	129	19	20	4.4	0.41 (0.17–0.98)
Country of birth					
Norway	323	0.8	52	0.2	0.27 (0.18–0.40)
Overseas	203	1.7	34	0.3	0.23 (0.14–0.38)
Unknown	26	4.4	5	1.4	2.33 (0.13–41.6)
Risk for severe COVID-19^b^					
No underlying comorbidities	297	0.7	36	0.1	0.23 (0.15–0.36)
Medium-risk comorbidity	145	2.8	31	0.9	0.35 (0.21–0.59)
High-risk comorbidity	110	13	24	5.1	0.16 (0.06–0.42)
Vaccination status at date of positive test					
Not vaccinated	335	1.5	15	0.2	0.13 (0.07–0.23)
One dose < 21 days before positive test	8	2.3	1	0.6	NA
Partially completed primary vaccination series ≥ 21 days before positive test^c^	9	0.2	3	0.1	NA
Completed primary vaccination series with maximum two doses 7–179 days before positive test^c, d^	47	0.3	26	0.1	0.62 (0.31–1.24)
Completed primary vaccination series with maximum two doses ≥ 180 days before positive test^c, d^	98	2.1	26	0.7	0.50 (0.25–1.01)
Vaccinated with three doses ≥ 7 days before positive test^c^	55	3.7	20	0.6	0.19 (0.08–0.43)
Unvaccinated, but previously diagnosed with COVID-19 6–12 months before positive test	0	0.0	0	0.0	NA^e^

CI: confidence interval; COVID-19: coronavirus disease; NA: not available due to small numbers and lack of discordant pairs; SARS-CoV-2: severe acute respiratory syndrome coronavirus 2.

^a^ Proportions here are calculated using as denominator the respective population in the subgroup and variant as reported in Table 1.

^b^ Risk for severe disease based on underlying comorbidities that are associated with a moderate or high risk of serious illness regardless of age. Details on the definitions used are provided in Supplement section 1.

^c^ See Supplement section 1 for full details on how each vaccination status category is defined and data on vaccine type. Overall, 98% of vaccinated Omicron cases and 96% of Delta cases had received a homologous or mixed regimen of mRNA vaccines. The three-dose category predominantly includes cases who received their third dose as a booster dose, however it will also include cases who received their third dose as part of their primary series, for example those severely immunocompromised [14]. We were not able to clearly distinguish persons who had received a third dose as part of their primary series from those who had received a booster dose.

^d^ When we used a recoded variable for vaccination status including both categories for those who had completed primary vaccination with maximum two doses, regardless of the time since last dose, we found an adjusted hazard ratio of 0.54 (95% CI: 0.34–0.87).

^e^ We had 415 cases who were unvaccinated, but who had previously been diagnosed with COVID-19 6–12 months before positive test. None of these 415 required hospitalisation. This indicates that prior infection is associated with lower risk of hospitalisation than unvaccinated status, but we could not calculate estimates because of a lack of discordant pairs in our model.

Hazard ratios were estimated using Cox regression stratified by county of residence and date of sampling and further adjusted for variant, sex, age group, country of birth, underlying comorbidities and vaccination status at date of positive test.

In Table 2, we present a subgroup analysis of the risk of hospitalisation following infection with the Omicron compared with the Delta variant. The aHR indicated a similar association (reduced risk), except for strata with a small number of cases and few hospitalisations and for some subgroups by vaccination status. We observed interactions between vaccination status and (i) variant, (ii) age group and (iii) underlying comorbidities. We chose not to include these interactions in the main analysis, and investigated them separately in a subgroup analysis by vaccination status (Table 3). In this subgroup analysis, the reduction in the risk of hospitalisation for Omicron compared with Delta was smaller among cases who had completed primary vaccination with maximum two doses 7–179 days before positive test compared with unvaccinated cases (66% for Omicron vs 93% for Delta with CI that did not overlap). For those vaccinated with three doses, the reduction in the risk of hospitalisation compared with unvaccinated cases was similar for Omicron and Delta cases (86% and 88% respectively with overlapping CI). However, among Omicron cases, we did not observe a significant decrease in risk for persons who had partially completed the primary vaccination series, nor persons who had completed primary vaccination with maximum two doses ≥ 180 days before positive test compared with unvaccinated.

**Table 3:** Subgroup analysis using stratified Cox regression for the risk of hospitalisation by vaccination status among COVID-19 cases, Norway, 6 December 2021–9 January 2022 (n = 91,005)

	Adjusted hazard ratio (95% CI) for hospitalisation					
	Not vaccinated	One dose < 21 days before positive test	Partially completed primary vaccination series ≥ 21 days before positive test^a^	Completed primary vaccination series with maximum two doses 7–179 days before positive test^a,b^	Completed primary vaccination series with maximum two doses ≥ 180 days before positive test^a,b^	Vaccinated with three doses ≥ 7 days before positive test^a^
Overall/main analysis	Ref	1.18 (0.59–2.39)	0.22 (0.12–0.41)	0.09 (0.07–0.12)	0.17 (0.14–0.22)	0.10 (0.07–0.14)
Subgroup analysis by						
Variant						
Delta	Ref	1.18 (0.56–2.50)	0.19 (0.09–0.38)	0.07 (0.05–0.10)	0.16 (0.12–0.21)	0.12 (0.09–0.17)
Omicron	Ref	2.03 (0.24–17.4)	0.71 (0.20–2.51)	0.34 (0.17–0.68)	0.59 (0.28–1.22)	0.14 (0.06–0.31)
Age group (years)						
0–29	Ref	5.72 (1.26–26.1)	0.16 (0.02–1.16)	0.45 (0.19–1.08)	1.53 (0.47–4.94)	0.57 (0.05–6.63)
30–44	Ref	NA	0.20 (0.05–0.84)	0.07 (0.04–0.13)	0.27 (0.13–0.55)	0.29 (0.10–0.84)
45–54	Ref	1.33 (0.28–6.28)	0.33 (0.08–1.42)	0.09 (0.05–0.16)	0.15 (0.07–0.31)	0.26 (0.11–0.60)
55–64	Ref	1.50 (0.15–14.5)	0.16 (0.02–1.22)	0.05 (0.03–0.10)	0.12 (0.07–0.20)	0.17 (0.08–0.34)
65–74	Ref	2.36 (0.31–18.2)	0.54 (0.11–2.74)	0.17 (0.07–0.42)	0.11 (0.06–0.21)	0.09 (0.04–0.18)
≥ 75	Ref	3.27 (0.41–25.8)	0.40 (0.08–1.88)	0.29 (0.10–0.83)	0.27 (0.15–0.50)	0.04 (0.02–0.09)
Risk for severe COVID-19^c^						
No underlying comorbidities	Ref	1.11 (0.50–2.44)	0.28 (0.14–0.58)	0.06 (0.04–0.09)	0.13 (0.08–0.19)	0.03 (0.01–0.07)
Medium-risk comorbidity	Ref	0.64 (0.08–5.05)	0.18 (0.04–0.79)	0.16 (0.10–0.26)	0.20 (0.14–0.30)	0.07 (0.04–0.12)
High-risk comorbidity	Ref	7.96 (0.41–155.7)	1.53 (0.16–14.4)	0.29 (0.08–1.11)	0.87 (0.36–2.08)	0.63 (0.26–1.56)

aHR: adjusted hazard ratio; CI: confidence interval; NA: not available due to small numbers and lack of lack of discordant pairs; COVID-19: coronavirus disease .

^a^ See Supplement section 1 for full details on how each vaccination status category is defined and data on vaccine type. Overall, 98% of vaccinated Omicron cases and 96% of Delta cases had received a homologous or mixed regimen of mRNA vaccines. The three-dose category predominantly includes cases who received their third dose as a booster dose, however it will also include cases who received their third dose as part of their primary series, for example those severely immunocompromised [14]. We were not able to clearly distinguish persons who had received a third dose as part of their primary series from those who had received a booster dose.

^b^ When we used a recoded variable for vaccination status including both categories for those who had completed primary vaccination with maximum two doses, regardless of the time since last dose, we estimated that Omicron cases had a 50% reduced risk (aHR = 0.50; 95% CI: 0.26–0.96) and Delta cases an 88% reduced risk of hospitalisation (aHR = 0.12; 95% CI: 0.09–0.14), respectively, compared with unvaccinated.

**^c^** Risk for severe disease based on underlying comorbidities that are associated with a moderate or high risk of serious illness regardless of age. Details on the definitions of medium- and high-risk categories are provided in Supplement section 1.

Hazard ratios for hospitalisation were estimated using Cox regression stratified by county of residence and date of sampling and further adjusted for variant, sex, age group, country of birth and underlying comorbidities at date of positive test.

**Figure 2:**
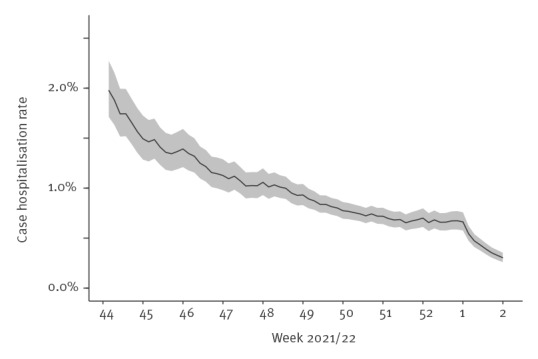
10-day average case COVID-19 hospitalisation rate (unadjusted) as a function of time, Norway, 1 November 2021– 9 January 2022 (n = 239,391)

In Figure 2, we present the 10-day average case hospitalisation rate (unadjusted) over time. The decrease in the rate in the early part of the plot corresponds to the ongoing roll-out of third doses, either as part of the primary series for persons with specific immunosuppressive conditions (from September 2021), or a booster dose (first offered to persons ≥ 65 years and care home residents from October 2021). We see a further decrease during late December and early January when the Omicron variant superseded Delta. This latter decrease is around 70%, indicating a population-level effect similar to the decrease observed in the study cohort. 

The figure shows the case hospitalisation rate by date of sampling. This rate is calculated as the 10-day averaged fraction of patients who are admitted to hospital (10-day average of hospitalised cases/reported cases by date of sampling). The calculation of case hospitalisation rate discounts the incidence of cases during the latest dates by the observed delay between testing and admission to account for potential right censoring [15].

## Length of stay in hospital and intensive care admission among hospitalised cases

At the end of follow-up, 10 of 91 (11%) patients infected with the Omicron variant and 80 of 552 (14%) patients infected with the Delta variant were still hospitalised. The crude median LoS among Omicron patients was 2.8 days (IQR: 1.6–6.8) compared with 6.5 (IQR: 3.2–12.3) among Delta patients. Seven Omicron patients (7.7%) were admitted to an ICU, compared with 135 (24%) Delta patients.

The aHR for discharge from hospital and risk of ICU admission for Omicron patients compared with Delta patients were calculated using Cox proportional hazard regression stratified for age, sex, vaccination status and number of underlying risk factors (see Supplement section 3 for detailed information on the statistical analysis). The aHR for discharge for Omicron patients compared with Delta patients was 1.44 (95% CI: 0.99–2.07). Assuming exponential distribution of the survival data, an aHR of 1.44 represents an expected 31% shorter LoS (95% CI: 1% longer–52% shorter). See Supplement section 4 for the fit of the LoS outcome to an exponential distribution. The aHR for the risk of ICU admission for Omicron patients compared with Delta patients was 0.51 (95% CI: 0.20–1.29).

## Reported deaths

Ten (seven non-hospitalised) and 92 (30 non-hospitalised) deaths were reported among the 39,524 Omicron and 51,481 Delta cases, respectively. Nine Omicron and 80 Delta deaths were reported as COVID-19 related (see Supplement section 1 for the definition of a COVID-19-related death). We did not further analyse data on deaths because the numbers were small.

## Ethical statement

Ethical approval was granted by Regional Committees for Medical and Health Research Ethics South East (reference number 249509). The need for informed consent was waived.

## Discussion

In this national register-based study, we found that reported COVID-19 cases infected with SARS-CoV-2 Omicron (subvariant BA.1) were associated with a 73% lower risk of hospitalisation compared with reported infection with the Delta variant. Our findings add to the growing evidence that people infected with Omicron have a lower risk of severe disease than those infected with Delta. Results presented in national reports from Denmark and the United Kingdom (UK), and studies from the United States and Canada estimated a 36–66% reduced risk of hospitalisation [5-8]. Our preliminary data on LoS and risk of ICU admission also indicate a probable milder disease trajectory among hospitalised Omicron patients than Delta patients, as reported by others [7]. However, our analysis for LoS and risk of ICU admission is based on a small cohort of hospitalised Omicron patients. Results for these outcomes and must be interpreted with caution at this early stage, and will require further investigation in larger patient cohorts.

We detected an interaction between variant and vaccination status. In the subgroup analysis, estimates for cases who had completed primary vaccination with maximum two doses 7–179 days indicated a lower protective effect of the vaccine for Omicron cases compared with Delta (66% and 93%, respectively). For other groups vaccinated with one or two doses, the estimates among Omicron cases were too uncertain to draw clear conclusions. The third dose of the vaccine was associated with a similar reduction in the risk of hospitalisation for Omicron and Delta compared with their respective unvaccinated cases (86% and 88%, respectively). These findings concur with reports from UK [6] and are expected, based on data from studies that estimated reduced vaccine effectiveness against Omicron infection [9-12] and the benefit of a booster dose [9,10].

During the study period, hospitals functioned within capacity, and the testing strategy in Norway was stable, with a high proportion of reported cases screened (59%) for variant. We stratified by sampling date and county of residence, considering differences in testing and screening by time and place. Although we noticed a slightly higher proportion of hospitalised cases being screened compared with non-hospitalised, we believe that this bias is limited. If we assume that we have oversampled hospitalised Delta cases, given the estimated higher risk of hospitalisation, this would cause us to slightly overestimate the reduction in the risk of hospitalisation for Omicron. Conversely, another potential bias could be systematic differences between the variants among non-diagnosed cases. For example, Omicron infections may have a higher rate of asymptomatic carriage than other variants of concern [13]. Such infections may be less likely to be diagnosed, which could result in an underestimation of the true reduction in risk of hospitalisation for Omicron compared with Delta.

## Conclusion

Information on severity of infection with new variants is central for decision-making on control measures and strategies. However, considering the increased transmissibility of the Omicron variant and the reduced vaccine effectiveness against Omicron infection, we need to consider that the lower risk of severe disease associated with Omicron does not necessarily imply a reduced burden on hospitals, especially in the event of large waves of infections during the winter season when other viruses also circulate.

## Supplementary Data

346000 Supplement
