# Supplementary material for: Reduced risk of hospitalisation among reported COVID-19 cases infected with the SARS-CoV-2 Omicron BA.1 variant compared with the Delta variant, Norway, December 2021 to January 2022
Source: Euro Surveill. 2022 Jan 27;27(4):2200077. doi: 10.2807/1560-7917.ES.2022.27.4.2200077 (PMC8796289; doi:10.2807/1560-7917.ES.2022.27.4.2200077)
Supplement: Supplement [file 22-00077_VENETI_Supplement.pdf]

Supplementary material: Reduced risk of hospitalisation among reported COVID-19 cases infected with the SARS-CoV-2 Omicron BA.1 variant compared with the Delta variant, Norway, December 2021 to January 2022

This supplementary material is hosted by Eurosurveillance as supporting information alongside the article "Reduced risk of hospitalisation among reported COVID-19 cases infected with the SARS-CoV-2 Omicron BA.1 variant compared with the Delta variant, Norway, December 2021 to January 2022" on behalf of the authors who remain responsible for the accuracy and appropriateness of the content. The same standards for ethics, copyright, attributions and permissions as for the article apply. Supplements are not edited by Eurosurveillance and the journal is not responsible for the maintenance of any links or email addresses provided therein.

1. Additional information on the data sources and definitions

All data in this study came from the national emergency preparedness register, Beredt C19. Beredt C19 contains individual-level data from central health registries, national clinical registries and other national administrative registries.

1.1 Reported cases of COVID-19 and COVID-19 related deaths

We included data on reported cases of laboratory-confirmed SARS-CoV-2 infection and COVID-19 related deaths from the Norwegian Surveillance System for Communicable Diseases (MSIS). As of January 2022, in MSIS, reinfections are registered if there are ≥6 months between two positive sampling dates for an individual, although this will exclude reinfections within a 6-month period, of which the Omicron variant could be of higher risk [1]. In Norway, COVID-19 related deaths are defined as deaths among COVID-19 cases notified to the Norwegian Institute of Public Health by a physician, or deaths where COVID-19 is reported on the death certificate (through linkage to the Cause of Death Registry). More details are available here (in Norwegian): https://www.fhi.no/sv/smittsomme-sykdommer/corona/dags--og-ukerapporter/sporsmal-og-svar-om-koronaovervaking-og-statistikk/.

1.2 Laboratory testing for variants

Data on virus variants came from the MSIS laboratory database (national laboratory database), which receives SARS-CoV-2 test results from all Norwegian microbiology laboratories. Variants are identified based on whole genome sequencing, Sanger partial S-gene sequencing or PCR screening targeting specific single nucleotide polymorphisms, insertions or deletions that reliably differentiate between omicron and other variants. The laboratory testing for variants of SARS-CoV-2 in Norway has been described in further detail elsewhere [2].

1.3 National identity number

Data on persons with a national identity number was drawn from the national population registry. The national identity number was essential to link data from all registries used in the analysis. The national population registry was also used to identify all deaths in our study cohort during the study period, regardless og whether they were COVID-19 related or not.

1.4 Admission to hospital and intensive care admission

We obtained data on hospitalisation following a positive SARS-CoV-2 test from the Norwegian Intensive Care and Pandemic Registry (NIPaR). All Norwegian hospitals report to NIPaR, and reporting is mandatory. Hospitals in Norway functioned within capacity during the study period, and criteria for hospitalisation and isolation for COVID-19 patients were consistent.

For patients who contracted COVID-19 while admitted to hospital, the time of admission is set to the date of symptom onset, or date of sampling if the patient is asymptomatic. The reported main cause of hospitalisation is a clinical assessment. For patients reported with a different main cause than COVID-19, we cannot rule out that COVID-19 may have been a contributing factor for admission. There is no reason to believe, however, that this assessment would differ between patients infected with different variants.

In NIPaR, underlying risk factors for severe COVID-19 diagnosed before admission are registered. The following risk factors are registered; asthma, cancer, chronic lung disease, chronic neurological or neuromuscular disease, diabetes (type 1 and 2), heart disease including hypertension, immunocompromised including HIV and immunosuppressive treatment (includes ongoing use of steroids in doses equivalent to at least 5mg Prednisolone daily), kidney disease including kidney failure, liver disease including liver failure, pregnancy, current smoker and body mass index (calculated as weight in kilograms divided by height in metres squared). For cancer, only active cancer is registered, meaning cancer where the patient still receives treatment, or regular control (>1 per year). Other well-regulated or treated conditions are not distinguished from unregulated or untreated conditions, for example asthma. For the analysis of length of stay (LoS) in hospital and risk of intensive care unit (ICU) admission we used data on risk factors from NIPaR, instead of the data on underlying comorbidities that was used for the analysis of reported cases (see 1.6). This is because the reporting of data on risk factors for hospitalised patients is more complete that the data in 1.6. Full details on the registration of hospitalised patients are available here (in Norwegian): https://helse-bergen.no/norsk-pandemiregister/registrering-i-norsk-pandemiregister-informasjon-til-ansatte

NIPaR also includes data on patients who have tested positive for COVID-19 and are admitted to an ICU. Patients are registered as ICU patients if they fulfil one of five categories:

1. LoS over 24 hours in intensive care
2. Require mechanical ventilation
3. Are transferred between intensive care wards
4. Persistent administration of vasoactive medication
5. LoS under 24 hours, but passed away during stay in intensive care

Full details on the registration of ICU patients are available here (in Norwegian): https://helse-bergen.no/norsk-pandemiregister/registrering-i-norsk-pandemiregister-informasjon-til-ansatte.

1.5 Vaccination status and vaccine type

Data on COVID-19 vaccinations came from the Norwegian Immunisation Registry, SYSVAK. In Norway, the mRNA vaccines Comirnaty® (BioNTech-Pfizer, Mainz, Germany/New York, United States) and Spikevax® (mRNA-1273, Moderna, Cambridge, United States) have been the two predominant vaccines administered. We defined COVID-19 cases according to the vaccination doses received at date of positive test:

1. Unvaccinated with a COVID-19 vaccine before positive test.
2. Vaccinated with 1 dose of a COVID-19 vaccine <21 days before positive test.
3. Partially completed primary vaccination series ≥21 days before positive test – those who tested positive ≥21 days after their first dose of a COVID-19 vaccine with a minimum two-dose primary series, and <7 days after the second dose.
4. Completed primary vaccination series with maximum two doses before positive test – those who tested positive ≥7 days after their second dose and <7 days after their third dose, with at least the

recommended minimum interval between doses depending on the type of vaccine (https://www.fhi.no/om/koronasertifikat/til-helsepersonell-vanlige-problemstillinger-om-koronasertifikat/#oversikt-over-intervall-mellom-koronavaksiner). This group also includes persons who had tested positive ≥7 days after their first vaccine dose if they had previously also been diagnosed with COVID-19 ≥21 days before vaccination and 6–12 months before their current positive test (n=1,116 for Omicron cases, 4.4% of Omicron cases with two doses; n=80 for Delta cases, 0.4%), and persons who had tested positive 6–12 months after a previously reported COVID-19 cases if they had received their first vaccine dose ≥21 days before the first reported case (n=7 for Omicron cases, 0,03%; n=1 for Delta cases, 0,004%). Cases who received the Janssen vaccine® (Janssen Vaccines, Leiden, Netherlands) were also included if they tested positive ≥21 days after one dose (n=71 for Omicron cases, 0,3%; n=100 for Delta cases, 0,4%). We further divided up this the two-dose category into those who had received their last dose 7–179 days before positive test, and those who had received their last dose ≥180 days before positive test.

5. Three doses – those who tested positive ≥7 days after their third dose. This category predominantly includes cases who received their third dose as a booster dose, however it will also include cases who received their third dose as part of their primary series, for example those severely immunocompromised (see https://www.fhi.no/nyheter/2021/flere-med-alvorlig-nedsatt-immunforsvar-bor-ta-3.-dose-koronavaksine/). We were not able to clearly distinguish persons who had received a third dose as part of their primary series from those who had received a booster dose.

We present the age and risk group distribution of cases in our study cohort by vaccination status in Table S1.

Among the 31,478 Omicron cases who had received at least one vaccine dose, 17,173 (55%) had received a homologous Comirnaty regimen, 7,158 (23%) had received a mix of Comirnaty and Spikevax and 6,180 (20%) had received a homologous Spikevax regimen. A further 522 (1.7%) had received a mix of Comirnaty or Spikevax with Astra Zeneca, and 373 (1.2%) had received a homologous Astra Zeneca regimen. The remaining 72 had received Janssen, or a mix of Janssen with Comirnaty or Spikevax.

Among the 28,566 Delta cases who had received at least one vaccine dose, 18,828 (66%) had received a homologous Comirnaty regimen, 4,933 (17%) had received a mix of Comirnaty and Spikevax and 3,726 (13%) had received a homologous Spikevax regimen. A further 603 (2.1%) had received a homologous Astra Zeneca regimen, and 374 (1.3%) had received a mix of Comirnaty or Spikevax with Astra Zeneca. The remaining 102 had received Janssen, Covishield or a mix of Janssen with Comirnaty or Spikevax.

Table S1: Characteristics of COVID-19 cases in the study cohort, by virus variant and vaccination status, Norway, 6 December 2021– 9 January 2022

	Delta variant cases (n=51,481), n (%)							Omicron variant cases (n=39,524), n (%)						
	Not vaccinated	One dose < 21 days before positive test	Partially completed primary vaccination series ≥ 21 days before positive test	Completed primary vaccination series with maximum two doses 7–179 days before positive test	Completed primary vaccination series with maximum two doses ≥ 180 days before positive test	Vaccinated with three doses ≥ 7 days before positive test	Unvaccinated, but previously diagnosed with COVID-19 6–12 months before positive test	Not vaccinated	One dose < 21 days before positive test	Partially completed primary vaccination series ≥ 21 days before positive test	Completed primary vaccination series with maximum two doses 7–179 days before positive test	Completed primary vaccination series with maximum two doses ≥ 180 days before positive test	Vaccinated with three doses ≥ 7 days before positive test	Unvaccinated, but previously diagnosed with COVID-19 6–12 months before positive test
**Total**	**22,837 (44%)**	**355 (0.7%)**	**3,935 (7.6%)**	**17,981 (35%)**	**4,790 (9%)**	**1,505 (2.9%)**	**78 (0.2%)**	**7,709 (20%)**	**162 (0.4%)**	**2,573 (6.5%)**	**21,840 (55%)**	**3,561 (9%)**	**3,343 (8.5%)**	**337 (0.9%)**
**Age group**														
0-29 years	17,787 (78%)	202 (57%)	3,409 (87%)	3,626 (20%)	465 (9.7%)	63 (4.2%)	54 (69%)	6,361 (82 %)	94 (58%)	2,266 (88%)	9,820 (45%)	940 (26%)	414 (12%)	272 (81%)
30-44 years	3,182 (14%)	111 (31%)	359 (9.1%)	8,329 (46%)	1,207 (25%)	303 (20%)	14 (18%)	940 (12%)	53 (33%)	243 (9.5%)	7,424 (34%)	995 (28%)	551 (16%)	50 (15%)
45-54 years	1,061 (4.7%)	26 (7%)	96 (2.4%)	4,086 (23%)	1,042 (22%)	220 (15%)	6 (7.7%)	2,447 (3.2%)	7 (4.3%)	39 (1.5%)	3,269 (15%)	842 (24%)	705 (21%)	11 (3.3%)
55-64 years	499 (2.2%)	6 (1.7%)	43 (1.1%)	1,768 (9.8%)	1,081 (23%)	222 (25%)	4 (5.1%)	117 (1.5%)	5 (3.1%)	15 (0.6%)	1,275 (5.8%)	591 (17%)	704 (21%)	4 (1.2%)
65-74 years	182 (0.8%)	5 (1.4%)	17 (0.4%)	145 (0.8 %)	774 (16%)	426 (28%)	0 (0%)	28 (0.4%)	2 (1.2%)	7 (0.3%)	39 (0.2%)	151 (4.2%)	592 (18%)	0 (0%)
≥75 years	126 (0.6%)	5 (1.4%)	11 (0.3%)	36 (0.2%)	221 (4.6%)	271 (18%)	0 (0%)	16 (0.2%)	1 (0.6%)	2 (0.1%)	13 (0.1%)	43 (1.2%)	377 (11%)	0 (0%)
**Risk for severe COVID-19 ***														
No underlying comorbidities	21,119 (92%)	331 (93%)	3,629 (92%)	16,278 (91%)	3,198 (67%)	862 (57%)	75 (96%)	7,200 (93%)	151 (93%)	2,368 (92%)	20,545 (94%)	2,680 (75%)	2.300 (69%)	300 (89%)
Medium risk comorbidity	1,603 (7.0%)	22 (6.2%)	290 (7.4%)	1,527 (8.5%)	1,264 (26%)	450 (30%)	3 (3.9%)	487 (6.3%)	11 (6.8%)	194 (7.5%)	1,215 (5.6%)	749 (21%)	816 (24%)	36 (11%)
High risk comorbidity	115 (0.5%)	2 (0.6%)	16 (0.4%)	176 (1.0%)	328 (6.9%)	193 (13%)	0 (0%)	22 (0.3%)	0 (0%)	10 (0.4%)	80 (0.4%)	132 (3.7%)	227 (6.8%)	1 (0.3%)

** Risk for severe disease based on underlying comorbidities that are associated with a moderate or high risk of serious illness regardless of age. Details on the definitions used are provided in the Supplement, section 1.*

1.6 Underlying comorbidities

Data on underlying comorbidities, as stipulated by the national COVID-19 vaccination programme, was based on ICD-10 codes from the Norwegian Patient Registry, and ICPC-2 codes from the Norway Control and Payment of Health Reimbursement database (Table S2). The underlying comorbidities that have been defined as increasing the risk of severe COVID-19 are divided into two groups.

Medium risk includes people with diseases/conditions that entail a moderate risk of severe COVID-19. This includes chronic liver disease or significant hepatic impairment, immunosuppressive therapy as in autoimmune diseases, diabetes, chronic lung disease including cystic fibrosis and severe asthma which have required the use of high dose inhaled or oral steroids within the past year, obesity with a body mass index (BMI) of ≥35 kg/m2, dementia, chronic heart and vascular disease (with the exception of high blood pressure) and stroke.

High risk includes people with diseases/conditions that carry a high risk of severe COVID-19, also in younger individuals. These comorbidities include having received an organ transplant, immunodeficiency, hematological cancer in the last five years, other active cancers, ongoing or recently discontinued treatment for cancer (especially immunosuppressive therapy, radiation therapy to the lungs or cytotoxic drugs), neurological or neuromuscular diseases that cause impaired cough or lung function (e.g., ALS and cerebral palsy), Down syndrome and chronic kidney disease, or significant renal impairment.

The method used to determine underlying comorbidities will likely underestimate the true prevalence, as only individuals that have been in contact with health services are identified. Data on medications used and procedure codes are currently not taken into account, which would improve the definitions and detect more individuals with underlying comorbidities.

Table S2. ICD-10 codes from the Norwegian Patient Registry and ICPC-2 codes from the Norway Control and Payment of Health Reimbursement database used to identify cases with underlying comorbidities.

Underlying comorbidity	Specifications	ICD10-codes	ICPC-2 codes
Cardiovascular diseases, not including hypertension		I05, I06, I07, I08, I09, I2, I31, I32, I34, I35, I36, I37, I39, I40, I41, I42, I43, I46, I48, I49, I50, I60, I61, I62, I63, I64, I69.1, I69.2, I69.3, I69.4, I69.8, I69.0	K74, K75, K76, K77, K78, K82, K83, K90, K91
Chronic pulmonary diseases, including asthma		J41, J42, J43, J44, J45, J46, J47, J84, J98, E84	R95, R96
Compromised immune function	Organ transplantation, immune deficiency disorders, autoimmune conditions treated with immunosuppressants	Z94.0, Z94.1, Z94.2, Z94.3, Z94.4, Z94.8, D80, D81, D82, D83, D84, G35, M05, M08, M06, M07, M09, M13, M14, K50, K51	
Neurological and musculoskeletal disorders with compromised lung or cough function		G1, G20, G21, G23, G24, G40.5, G61.0, 70, G71, G80.0, G80.2, G80.3, F72, F73, F84.0, F84.1, Q05.0, Q05.1, Q05.2, Q05.3, Q05.04, Q05.5, Q05.6	

Diabetes		E10, E11, E12, E13, E14	T89, T90
Active cancer treatment or hematological cancer		C81, C82, C83, C84, C85, C86, C87, C88, C89, C90, C91, C92, C93, C94, C95, C96, D45, D45, D47, C0, C1, C2, C3, C4, C5, C6, C7, C80, D32, D33, D35.2, D35.3, D35.4, D42, D43, D44.2, D44.3, D44.4	
Other risk groups	Dementia, chronic kidney and liver disease, obesity	N18.3, N18.4, N18.5, K70.4, K72, F00, F01, F02, F03, G30, G31, E66	P70, T82

2. Assessment of representativeness of study population

We assessed the representativeness of our study population by comparing the characteristics of cases who were screened for the SARS-CoV-2 variant that they were infected with, with those who were not. Of 155,388 cases diagnosed in the study period with a national identity number, 91,772 (59%) were screened. We found differences between cases who were screened for variants with regards to county of residence, sampling week, hospitalisation, age and vaccination status (Table S3).

Differences in county reflect the differing capacity to screen for variants at different laboratories around Norway, while variant data were available for a lower proportion of cases in week 1 2022 (48%) which will likely increase as more variant results for cases reported in this week are registered. The proportion of cases screened among hospitalised cases was higher than among those not hospitalised (73% vs 59%). This reflects the national recommendation to prioritise screening of hospitalised COVID-19 patients, particularly when laboratory capacity is stretched. Furthermore, among hospitalised cases a larger proportion of patients admitted to ICU were screened compared to those who were not admitted to ICU (81% vs 72%). Therefore, our hospitalised cohort with known variant is slightly more representative of COVID-19 patients that require intensive care treatment, while our cohort of cases is slightly more representative of those who require hospital admission. This may explain some of the small differences we see in the proportion of cases screened in different age groups and vaccination status. However, given the small proportion of all cases admitted to hospital and that screening of hospitalised cases did not depend on exposure to Delta or Omicron, we do not consider that this would have notably influenced our estimates. The proportion of cases screened by age group varied from 56% – 62%, and was higher among the age groups 30–89 years. The proportion of cases screened by vaccination status varied from 55% – 64%, and was higher among the those who had completed primary vaccination with maximum two doses ≥180 days before positive test.

Table S3: Characteristics of reported cases by whether or not they were screened for the SARS-CoV-2 variant that they were infected with, 6 December 2021- 9 January 2022, Norway.

Characteristics		Screened for variant	
		No (n=63616)	Yes (n=91772)
Sex	Female	31524 (40.9%)	45638 (59.1%)
	Male	32092 (41.0%)	46134 (59.0%)
			p = 0.494
Age group	0-9 year	10241 (43.8%)	13163 (56.2%)
	10-19 year	12289 (41.5%)	17357 (58.5%)
	20-29 year	10911 (41.2%)	15547 (58.8%)
	30-39 year	10649 (39.7%)	16188 (60.3%)

	40-49 year	9514 (39.9%)	14306 (60.1%)
	50-59 year	6011 (40.0%)	9006 (60.0%)
	60-69 year	2668 (39.5%)	4089 (60.5%)
	70-79 year	955 (38.4%)	1529 (61.6%)
	80-89 year	283 (37.8%)	465 (62.2%)
	≥90 year	95 (43.8%)	122 (56.2%)
			p < 0.001
Median age	In years (interquartile range)	28 (13-43)	29 (14-44)
			p < 0.001
County of residence	Agder	2796 (26.9%)	7583 (73.1%)
	Innlandet	2559 (41.2%)	3653 (58.8%)
	Møre og Romsdal	2671 (80.5%)	649 (19.5%)
	Nordland	2274 (86.4%)	357 (13.6%)
	Oslo	9595 (25.8%)	27556 (74.2%)
	Rogaland	3246 (25.3%)	9573 (74.7%)
	Troms and Finnmark	2901 (77.6%)	838 (22.4%)
	Trøndelag	2795 (36.2%)	4925 (63.8%)
	Vestfold and Telemark	3320 (30.5%)	7578 (69.5%)
	Vestland	9939 (75.8%)	3172 (24.2%)
	Viken	21496 (45.4%)	25859 (54.6%)
	Unknown	24 (45.3%)	29 (54.7%)
			p < 0.001
Country of birth	Norway	46714 (41.1%)	66987 (58.9%)
	Overseas	16200 (40.5%)	23801 (59.5%)
	Unknown	702 (41.6%)	984 (58.4%)
			p = 0.103
Risk for severe COVID-19	No underlying comorbidities	56796 (41.0%)	81628 (59.0%)
	Medium-risk comorbidity	6260 (40.3%)	9255 (59.7%)
	High-risk comorbidity	560 (38.6%)	889 (61.4%)
			p = 0.053
Vaccination status	Not vaccinated	21366 (41.0%)	30744 (59.0%)
	One dose <21 days before positive test	347 (40.0%)	521 (60.0%)
	Partially completed primary vaccination series ≥21 days before positive test	4696 (41.7%)	6562 (58.3%)
	Completed primary vaccination series with maximum two doses 7–179 days before positive test	28043 (41.1%)	40125 (58.9%)
	Completed primary vaccination series with maximum two doses ≥ 180 days before positive test	4803 (36.2%)	8454 (63.8%)
	Vaccinated with three doses ≥7 days before positive test	4063 (45.1%)	4946 (54.9%)
	Unvaccinated, but previously diagnosed with COVID-19 6–12 months before positive test	298 (41.5%)	420 (58.5%)
			p < 0.001
Week of positive test	2021-49	12020 (36.1%)	21237 (63.9%)
	2021-50	10133 (33.4%)	20160 (66.6%)
	2021-51	9063 (41.4%)	12835 (58.6%)
	2021-52	9287 (36.6%)	16066 (63.4%)
	2022-01	23113 (51.8%)	21474 (48.2%)
			p < 0.001
	Yes	352 (27.0%)	954 (73.0%)

Admitted to hospital	No	63264 (41.1%)	90818 (58.9%)
			p < 0.001
Among cases admitted to hospital			
Admitted to hospital with Covid-19 as main cause	Yes	228 (25.7%)	658 (74.3%)
	No	122 (29.5%)	292 (70.5%)
	Unknown	2 (33.3%)	4 (66.7%)
			p = 0.345
Admitted to intensive care	Yes	38 (18.7%)	165 (81.3%)
	No	314 (28.5%)	789 (71.5%)
			p = 0.004

P values calculated using chi-squared tests or Wilcoxon rank sum tests as appropriate

3. Statistical analysis

3.1 Risk of hospitalisation

Using stratified Cox proportional hazard regression, we estimated the risk (adjusted hazard ratio, aHR) of hospitalisation for Omicron compared to Delta. The models were stratified by county of residence and sampling date, and further adjusted for age group, sex, country of birth, underlying comorbidities and vaccination status. The stratification allows for the impact of the covariates to be non-proportional among levels, and for each level of a factor to have their own baseline hazard rate. Interactions were observed in our main analysis between the variant and vaccination status, age group and vaccination status, and underlying comorbidities and vaccination status. We chose not to include them in our main model and investigated them separately in a subgroup analysis by vaccination status that is presented in the table 2 in the article.

Time at risk: For hospitalised cases we used the duration of time (in days) from date of positive test to hospitalisation. For non-hospitalised cases we used as time at risk, the time from date of positive test to the day we extracted the data that were used (20 January) allowing 10 days follow up. We also censored the two cases that died without being hospitalised as at risk until the date of death. We replaced the time at risk to 0.5 days for hospitalised cases who tested positive 1–2 days after hospitalisation, or on the day of admission (n=226, of which 15 admitted 1–2 days before positive test).

We present the results from our main univariate and multivariable analysis in Table S4. This will allow the readers to see the estimates for hospitalisation for the rest of the variables that were not mentioned in the main article.

Statistical analysis was performed in Stata version 16 (Stata Corporation, College Station, Texas, US).

Table S4. Hazard ratio estimates for hospitalisation from univariate and multivariable Cox regression stratified by county of residence and date of sampling and further adjusted for variant, sex, age group, country of birth, underlying comorbidities, and vaccination status at date of positive test, Norway, 6 December 2021 – 9 January 2022.

		Hospitalisation			
		No	Yes (%)	Crude hazard ratio (95%CI)	Adjusted hazard ratio (95%CI)
Variant	Delta	50,929	552 (1.1 %)	Ref	Ref
	Omicron	39,433	91 (0.2 %)	0.22 (0.17-0.27)	0.27 (0.20-0.36)
Sex	Female	44,981	281 (0.6 %)	Ref	Ref

	Male	45,381	362 (0.8 %)	1.28 (1.09-1.49)	1.24 (1.05-1.47)
Age group	30-44 years	23,643	109 (0.5 %)	Ref	Ref
	0-29 years	45,726	47 (0.1 %)	0.22 (0.16-0.31)	0.11 (0.08-0.16)
	45-54 years	11,558	98 (0.8 %)	1.83 (1.40-2.41)	2.14 (1.61-2.83)
	55-64 years	6,201	133 (2.1 %)	4.60 (3.57-5.93)	4.68 (3.57-6.14)
	65-74 years	2,261	107 (4.5 %)	10.00 (7.66-13.06)	8.39 (6.19-11.38)
	≥75 years	973	149 (13 %)	31.18 (24.35-39.91)	22.55 (16.60-30.65)
Country of birth	Norway	66,113	375 (0.6 %)	Ref	Ref
	Overseas	23,331	237 (1.0 %)	1.79 (1.52-2.11)	1.32 (1.10-1.59)
	Unknown	918	31 (3.3 %)	5.87 (4.07-8.46)	0.69 (0.46-1.05)
Risk for severe COVID-19 [a]	No underlying comorbidities	80,703	333 (0.4 %)	Ref	Ref
	Medium risk comorbidity	8,902	223 (2.4 %)	6.00 (5.07-7.11)	2.75 (2.25-3.36)
	High risk comorbidity	757	87 (10 %)	26.33 (20.80-33.34)	8.93 (6.71-11.88)
Vaccine status at date of positive test	Not vaccinated	30,196	350 (1.2 %)	Ref	Ref
	One dose <21 days before positive test	508	9 (1.7 %)	1.52 (0.79-2.95)	1.18 (0.59-2.39)
	Partially completed primary vaccination series ≥21 days before positive test	6,495	12 (0.2 %)	0.16 (0.09-0.29)	0.22 (0.12-0.41)
	Completed primary vaccination series with maximum two doses 7–179 days before positive test	39,748	73 (0.2 %)	0.16 (0.12-0.21)	0.09 (0.07-0.12)
	Completed primary vaccination series with maximum two doses ≥180 days before positive test	8,227	124 (1.5 %)	1.30 (1.06-1.60)	0.17 (0.14-0.22)
	Vaccinated with three doses ≥7 days before positive test	4,773	75 (1.6 %)	1.36 (1.06-1.75)	0.10 (0.07-0.14)
	Unvaccinated, but previously diagnosed with COVID-19 6–12 months before positive test	415	0 (0.0 %)	NA [b]	NA [b]

[a] *Risk for severe disease based on underlying comorbidities that are associated with a moderate or high risk of serious illness regardless of age. Details on the definitions used are provided in the Supplement, section 1.*

[b] *NA: not available. We had 415 cases who were unvaccinated, but who had previously been diagnosed with COVID-19 6–12 months before positive test. None of these 415 required hospitalisation. This indicates that prior infection is associated with lower risk of hospitalisation than unvaccinated status, but we could not calculate estimates because of a lack of discordant pairs in our model.*

3.2 LoS in hospital and risk of ICU admission

We calculated LoS in hospital as the time between first admission and last discharge from hospital, and time to ICU admission as the time between first admission to hospital and first admission to ICU. For patients with >1

registered hospital stay, we included the time between consecutive stays if <24 hours. Separate stays were registered if a patient was discharged and readmitted, or transferred between wards or hospitals. Patients with unknown date of discharge from their last stay were considered to still be hospitalised. We did not restrict admissions by LoS.

We estimated the aHR for discharge and risk of ICU admission in a Cox proportional hazard model stratified for age (using the same categories as in the analysis for risk of hospitalisaiton), sex (male and female), vaccination status (categories: unvaccinated or vaccinated with one dose, completed primary vaccination series with maximum two doses 7–179 days before positive test, completed primary vaccination series with maximum two doses ≥180 days before positive test, three doses), and number of underlying risk factors (categories: 0, 1, 2, 3+). See part 1 for detailed information on the registration of underlying risk factors in NIPaR. We did not further adjust by variables such as hospital or date of admission due to the size of our hospitalised cohort and the short study period. Patients still hospitalised were censored.

For the LoS outcome, as hazard rates are not explicitly estimated in Cox regression, we also estimated a proxy for the expected difference in LoS as 1-(1/aHR), by assuming an exponential survival distribution (see part 4).

The statistical analysis was performed in R version 3.6.2.

4. Fit of LoS outcome to an exponential distribution

For LoS, as hazard rates are not explicitly estimated in Cox regression, we estimated a proxy for the expected difference in LoS as 1-(1/aHR), by assuming a constant baseline hazard rate, i.e. an exponential survival distribution. Figure S1 shows the fit of LoS data to an exponential distribution. The observed data differ notably from the expected distribution from approximately LoS >35 days (15 patients, 2.3% og all hospitalised patients in the study cohort), where the LoS was shorter than would be expected if all observations had followed an exponential distribution. All these 15 were infected with Delta. Thus, the estimated proportional decrease in overall LoS among patients infected with Omicron presented in the study is likely fractionally overestimated.

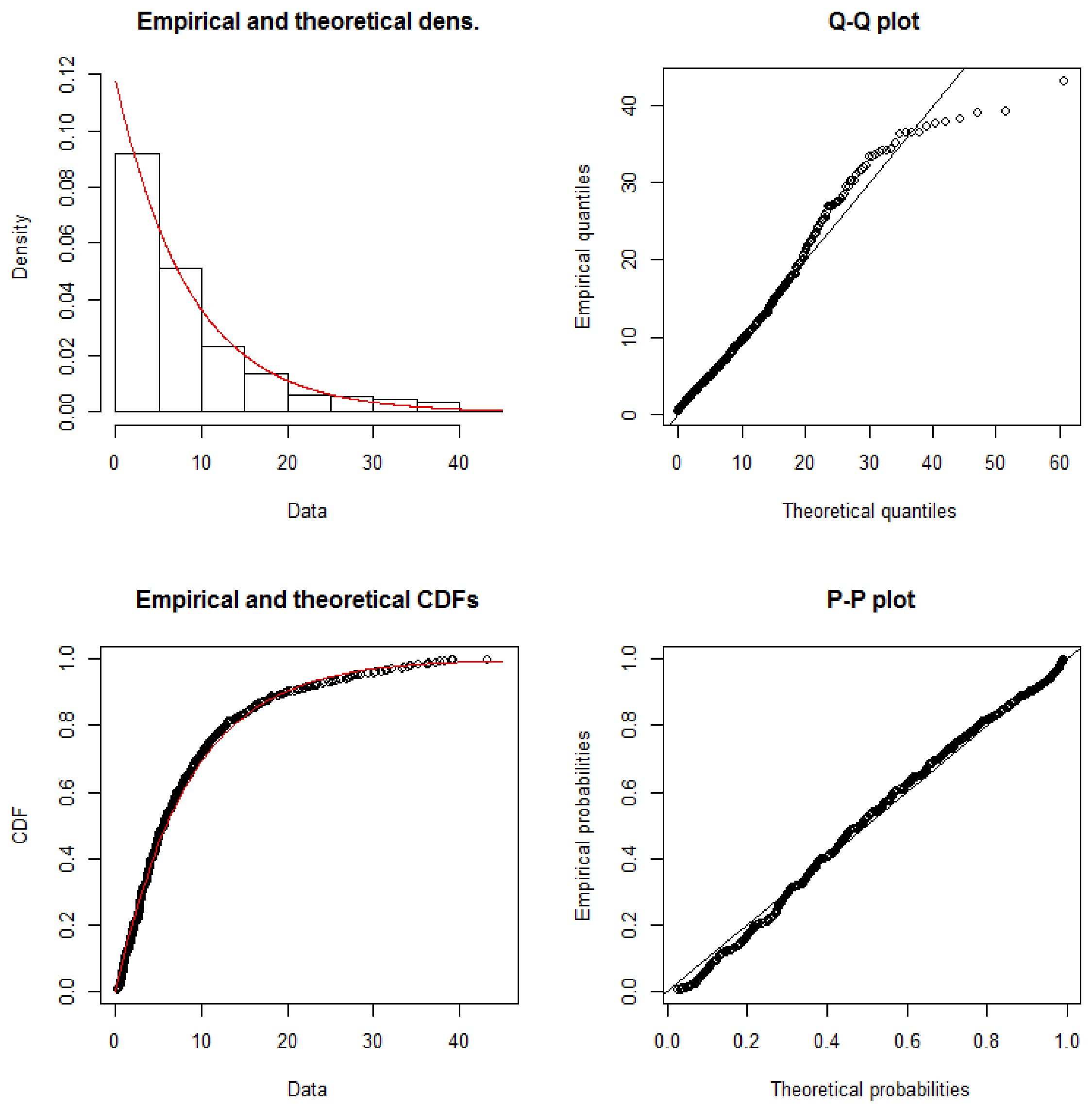

Figure S1. Fit of LoS in hospital to an exponential distribution (n=643).

5. References

1. Pulliam JRC, van Schalkwyk C, Govender N, von Gottberg A, Cohen C, Groome MJ, et al. Increased risk of SARS-CoV-2 reinfection associated with emergence of the Omicron variant in South Africa. Medrxiv. 2021. doi: https://doi.org/10.1101/2021.11.11.21266068

2. Norwegian Institute of Public Health. Påvisning og overvåkning av SARS-CoV-2-virusvarianter. Oslo: Norwegian Institute of Public Health. 2021 [cited 2022 Jan 20]. Available at: https://www.fhi.no/nettpub/coronavirus/testing/pavisning-og-overvakning-av-sars-cov-2-virusvarianter/
